# Neurovascular changes of the retina and optic nerve head in episodic migraine

**DOI:** 10.1038/s41598-024-71388-1

**Published:** 2024-08-30

**Authors:** Ágnes Patzkó, Zoltán Pfund, Adrienne Csutak, Noémi Tóth, Zsófia Kölkedi, Gréta Kis-Jakab, Edit Bosnyák, Renáta Rozgonyi, Eszter Szalai

**Affiliations:** 1 Department of Ophthalmology, University of Pécs Medical School, Rákóczi U. 2, Pécs, 7623 Hungary; 2 Department of Neurology, University of Pécs Medical School, Pécs, Hungary; 3 HUN-REN–PTE Clinical Neuroscience MR Research Group, Department of Neurosurgery, Medical School, University of Pécs, Pécs, Hungary

**Keywords:** Migraine, Optical coherence tomography angiography, Optic nerve head, Neurovascular, Medical research, Neurology, Pathogenesis

## Abstract

To investigate neurovascular changes; including macular vascular density (VD), thickness of the ganglion cell layer (GCL) and optic nerve head (ONH) parameters in episodic migraine patients. 80 eyes of 40 episodic migraine patients were recruited. Thirty patients having a dominant side of migraine headache were statistically analyzed (5 male and 25 female; mean age 31.67 ± 9.54 years) and compared to 25 eyes of 25 healthy volunteers (5 male and 20 female; mean age of 34.4 ± 12.11 years, *p* = 0.361). The posterior segment was imaged with Topcon DRI optical coherence tomography (OCT) (Triton Swept source OCT Topcon, Japan), and OCT angiography (OCTA). Comparing the dominant side of migraine patients to controls we found a significant decrease of the VD in the central zone of the superficial and deep capillary plexus (SCP, *p* = 0.01; DCP, *p* = 0.004) and an enlarged foveal avascular zone (FAZ, *p* = 0.054). The GCL thickness was significantly reduced in the central ring (GCL + *p* = 0.042, GCL +  + *p* = 0.029), as well as the retinal nerve fiber layer (RNFL) thickness in the temporal quadrant (*p* = 0.021) and border tissue of Elschnig diameter (BTE, *p* = 0.035). The duration of migraine showed an inverse correlation with SCP in the nasal quadrant (*p* = 0.016, r = − 0.445) and with all DCP regions [DCP superior (*p* = 0.004, r = − 0.519), DCP inferior (*p* = 0.004, r = − 0.519), DCP nasal (*p* = 0.006, r = − 0.496), DCP temporal (*p* = 0.005, r = − 0.508), DCP CSF (*p* < 0.001, r = − 0.634)]. The dominant side compared to the non-dominant side showed a significant deterioration of the VD in the inferior (*p* = 0.04) and temporal quadrants (*p* = 0.023); furthermore, a significant decrease in the GCL +  + inner ring thickness (*p* = 0.046). Microvascular damage and consequent structural alterations of the retina and optic nerve head occur in the eyes of episodic migraine patient in association with the lateralization of the headache.

## Introduction

Migraine is a multifaceted primary headache disorder with neurovascular involvement. Typically, it presents with a throbbing pain that can begin on one side of the head, commonly associated with nausea and vomiting, photo-, and phonophobia. According to the International Classification of Headache Disorders (ICHD) III, the unilateral presentation is one of the major diagnostic criterion for migraine^1^. Migraine is listed as the leading cause of years lived with disability in people under 50 years of age, affecting more than 1 billion patients worldwide^2,3^. Nearly 30% of migraine patients experience auras, a transient cortical malfunction leading to neurological symptoms^4^. Migraine is known to be accompanied with an increased risk of deep white matter lesions, silent posterior circulation territory infarcts, and infratentorial hyperintense lesions^5^. Not only the association with stroke but also the link to coronary heart disease has been extensively investigated. However, due to the low occurrence of these severe complications, to date it is not conceivable to ascertain which migraine patients will develop a cerebrovascular or cardiovascular event^6^.

Based on the vasospastic etiology of migraine with and without aura, several authors have lately described an alteration in the circulation of the retina, choroid and optic nerve and potential neurodegenerative consequences^7–9^. Optical coherence tomography angiography (OCTA)—a novel, noninvasive technique, that provides a direct visualization and quantification of the retinal microvasculature, has been widely used to evaluate the fundus of patients with neurological disorders^10^. The purpose of the present study was to investigate neurovascular changes including macular vascular density (VD), thickness of the ganglion cell layer (GCL) and optic nerve head (ONH) parameters including Bruch’s membrane opening (BMO), anterior scleral canal opening (ASO) and border tissue of Elschnig (BTE) in episodic migraine patients with and without aura and white matter hyperintensities (WMH). We wished to explore whether within-subject differences are present between the two eyes in the above mentioned parameters depending on the lateralization of the headache in episodic migraine patients with unilateral pain.

## Patients and methods

All migraine patients included in the study were admitted to the Outpatient Headache Department of the Department of Neurology, Medical School, University of Pécs, Hungary between July 2022 and April 2023 and fulfilled the definition of episodic migraine (International Headache Society (3rd edition)^1^. Informed consent was obtained from all subjects. All participants were evaluated neurologically in detail including history taking, physical examination, blood pressure measurement, serum and urine tests and a brain MRI study; migraine type, dominantly affected side, disease duration and attack frequency were determined as well. Patients were divided into 3 categories regarding disease duration (0–10 years, 11–20 years, > 20 years). Likewise, 3 groups were determined concerning monthly attack frequency (rare: 0–1 attacks/month, average: 2–7 attacks/month, very frequent: 8–15 attacks/month). For the ophthalmological examination patients who did not have any major comorbidities that could affect retinal circulation or optic nerve head parameters, such as hypertension, cardiac disease, diabetes, thyroid gland dysfunction, oncological and hematological diseases, infectious diseases (e.g. HIV, hepatitis), central nervous system demyelination (e.g. multiple sclerosis) were chosen carefully. Up to the time point of the ophthalmological examination 31 patients underwent a brain MRI examination performed with an 3 T MR scanner (Siemens Trio Tim, 12 channel head coil). The presence of white matter hyperintensities was assessed by a qualified neuroradiologist who was blinded to the diagnosis of migraine. WMH were defined as a hyperintensity, larger than 3 mm, visible on T2- weighted and FLAIR images, without hypointensity on T1- weighted scans^11^.

A comprehensive ophthalmologic examination was carried out including visual acuity, intraocular pressure, slit-lamp examination with fundus analysis. Patients with intraocular pathology, or surgery; furthermore, with myopic or hyperopic spherical equivalent refractive errors greater than 3.00 diopters were excluded.

The posterior segment was imaged with Topcon DRI optical coherence tomography (OCT) (Triton Swept source OCT Topcon, Japan), and OCT angiography (OCTA).

OCTA imaging—centered at the fovea with an automated layer segmentation for the superficial capillary plexus (SCP) and deep capillary plexus (DCP) using the built-in software segmentation algorithm (IMAGEnet 6 Version 1.26.16898, Topcon)—was carried out as described previously^12^. The superficial capillary plexus (SCP) was delineated by 2.6 µm below internal limiting membrane to 15.6 µm below the junction between inner plexiform layer (IPL) and inner nuclear layer (INL), for deep capillary plexus (DCP), 15.6 µm below IPL/INL to 70.2 µm below IPL/INL. The foveal avascular zone (FAZ) was manually outlined by the same observer (PA). For the structural OCT, SMARTTrack HD Raster centered to the macula (3.0 × 3.0 mm) was applied and 3D Disc program (6.0 × 6.0 mm, with a resolution of 512 × 256) was performed and centered at the optic nerve head. Retinal nerve fiber layer thickness (RNFL) in four quadrants and ganglion cell complex thickness (GCL + : between RNFL/GCL-inner plexiform layer/inner nuclear layer interface, GCL +  + : between inner limiting membrane—inner plexiform layer/inner nuclear layer interface) in three concentric zones were analyzed using the automated segmentation OCT map. Optic nerve head rim area (ONHRA), disc area (DA), linear (lCDR) and vertical cup-to-disc ratio (vCDR) and cup volume (CV) were also quantified by the software.

**Fig. 1:**
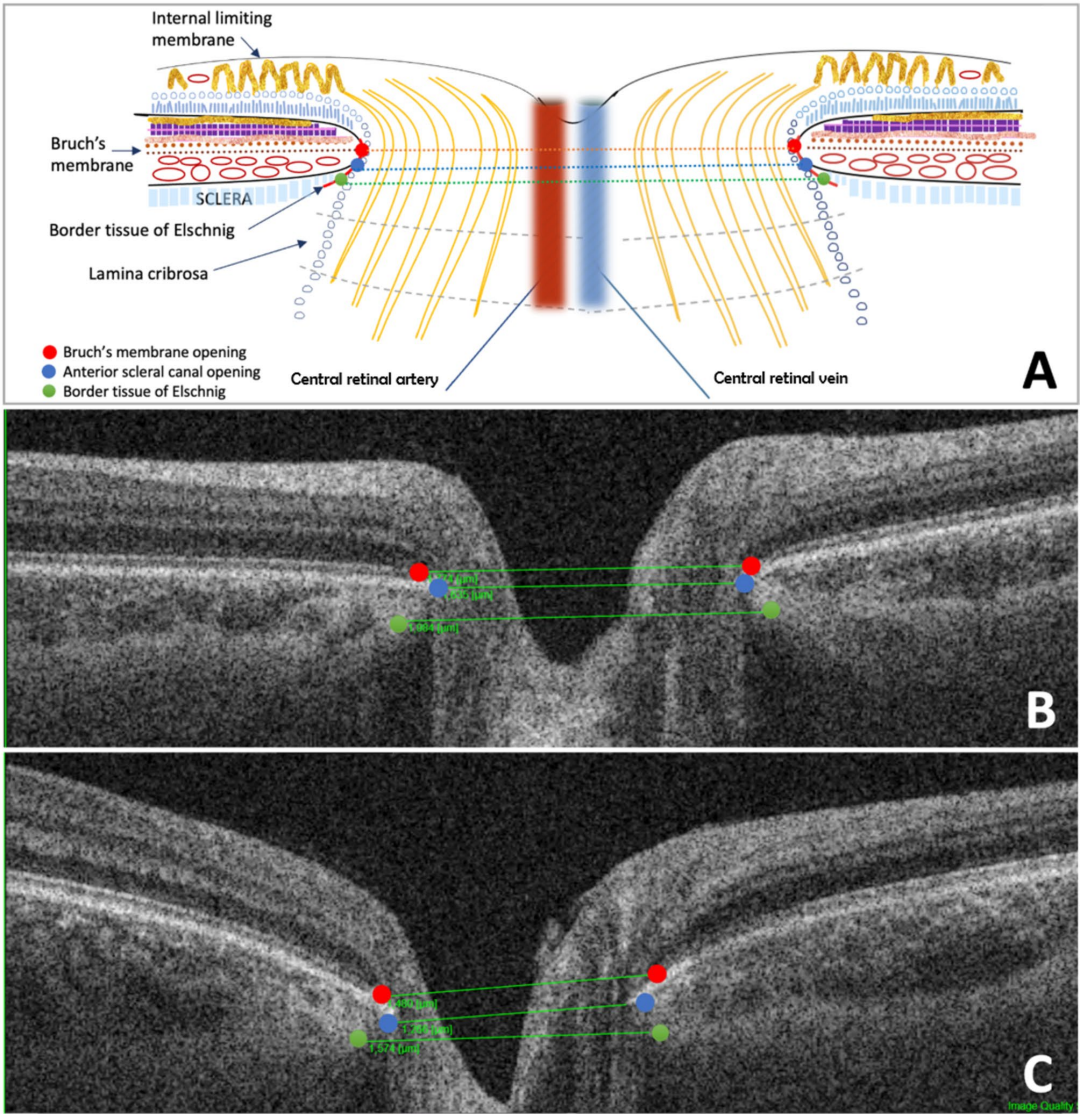
Structure of the bordering tissues of the optic nerve head (**A**) Schematic image; measurements on (**B**) control and (**C**) on the dominant side of episodic migraine patients (red: BMO, blue: ASO, green: BTE).

Bruch’s membrane opening (BMO), anterior scleral canal opening (ASO) and border tissue of Elschnig (BTE) values were measured in the central part of the optic disc. We slightly modified the delineation technique described by Strouthidis et al. in 2009 [7–8] (Fig. 1). Quantification of these structures was performed manually by using the built-in measuring tool of the OCT device. BMO was defined as the innermost extent of the posterior surface of the retinal pigment epithelium—Bruch’s membrane complex. ASO was demarcated as the internal boundary of the anterior scleral surface. BTE was determined as the junction of the choroid and the neural canal just below the RPE/BM complex^13^. Delineation of the ONH parameters in both study groups was performed by the same examiner (AP).

Control subjects had neither a history of any of the above mentioned systemic diseases nor an ocular diseases or surgery and underwent the same ophthalmological examinations as migraine patients.

The study was conducted in accordance with the tenets of the Helsinki Declaration and the protocol was approved by the University of Pécs Institutional Ethical Review Board (Number: 9535-PTE 2023).

### Statistical analysis

Data were analyzed using the SPSS Statistics 25.0 (IBM Corp., Armonk, NY). Mean, standard deviation (SD) and 95% confidence interval (95% CI) for the mean were calculated for each data set. The non-parametric Mann–Whitney U test was carried out to compare data of the dominantly affected side of migraine patients to controls; furthermore, to analyze differences between migraine patients with or without aura and WMH. For bivariate correlation analysis, the Spearman’s rank correlation “r” was used. The dominantly affected side of a migraine patient was compared to the contralateral side using Wilcoxon test. The analyzed eye of the controls was randomly selected. A *P* value below 0.05 was considered statistically significant.

## Results

80 eyes of 40 patients suffering from episodic migraine (6 male and 34 female) with a mean age of 31.75 ± 10.74 years (range: 18–59 years) were enrolled in our study. The dominantly affected side was the right side in 22 patients, the left side in 8 patients, and 10 patients showed bilateral involvement. Thirty patients having a dominant side (5 male and 25 female; mean age 31.67 ± 9.54 years, range: 18–49 years) were included in the further statistical analyzes and compared to 25 randomly selected eyes of 25 healthy volunteers (5 men and 20 women) with a mean age of 34.4 ± 12.11 years (range: 18–59 years) (*p* = 0.361). All 30 patients suffered from episodic migraine with an average disease duration of 14.45 ± 10.93 years and a monthly attack frequency of 4.21 ± 3.70. Twelve patients experienced a visual aura on a regular basis and 6 patients demonstrated with WMHs on MRI scans.

**Fig. 2:**
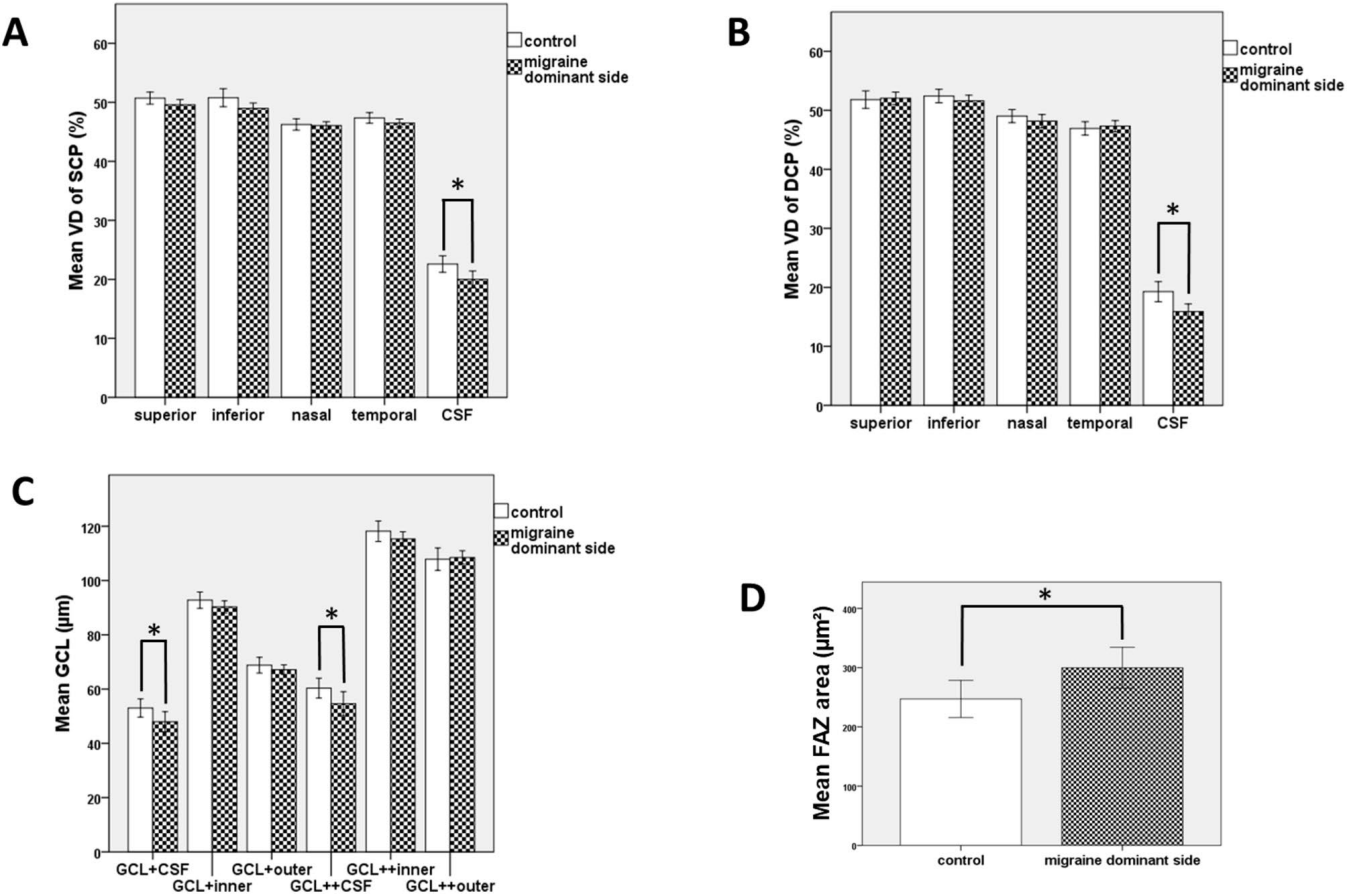
Changes in macular parameters observed on the dominant side of episodic migraine patients compared to controls. (**A**) significant decrease in the VD of CSF SCP; B as well as in the VD of CSF DCP; parallel with the **C**) significant reduction of the GCL + CSF and GCL +  + CSF; (**D**) significantly enlarged FAZ (*p ≤ 0.005).

**Fig. 3:**
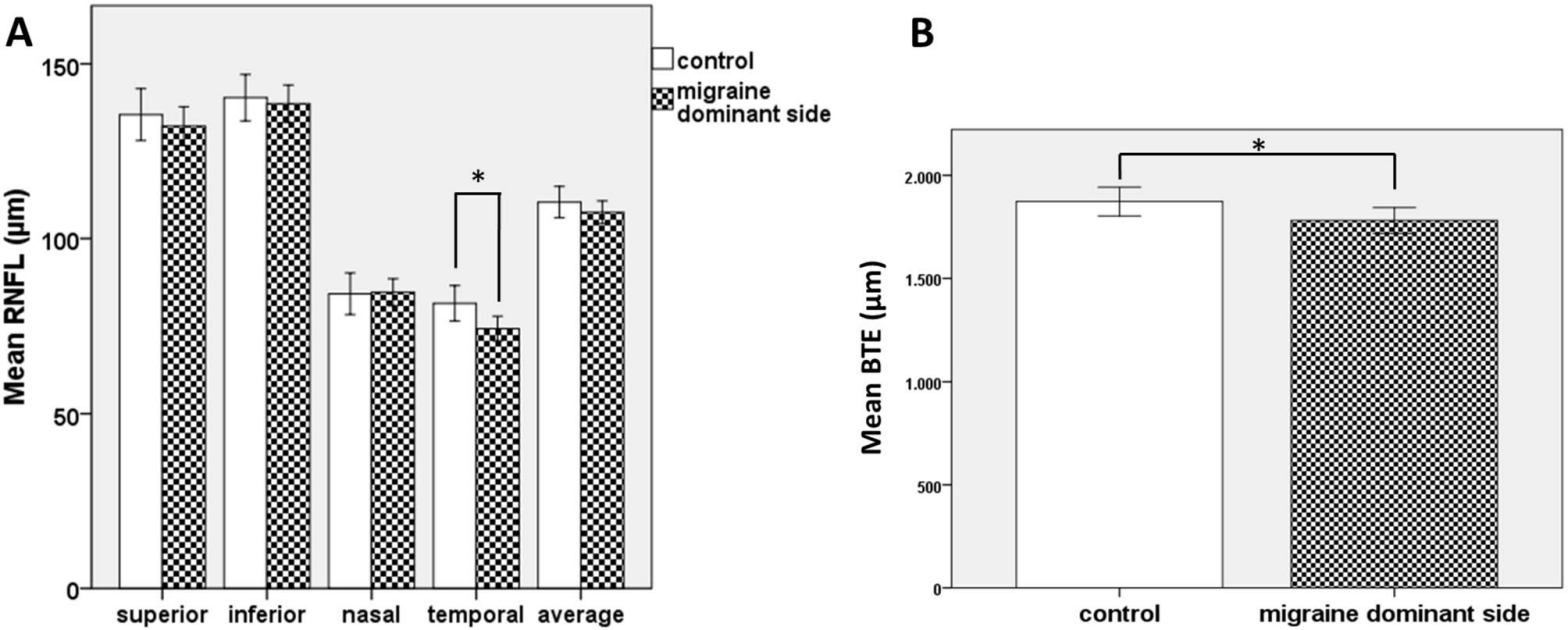
Alteration of optic nerve head parameters on the dominant side of episodic migraine patients compared to controls. (**A**) significant diminution of RNFL in the temporal quadrant; (**B**) significantly smaller BTE diameter (*p ≤ 0.005).

Comparing the dominant side of migraine patients to controls several alterations were detected in the macular microvasculature and optic nerve head parameters (Table 1, Figs. 2 and 3). A significant decrease of the vessel density in the central zone of the superficial plexus (*p* = 0.01) as well as in the central zone of the deep plexus (*p* = 0.004) were shown (Fig. 2). Regarding the superior, nasal, inferior and temporal quadrants, no significant alteration could be found. Moreover, the foveal avascular zone (FAZ) was significantly enlarged (*p* = 0.04; Fig. 2). The ganglion cell complex thickness was also significantly reduced in the central ring (GCL + *p* = 0.042, GCL +  + *p* = 0.029; Fig. 2). The duration of migraine showed an inverse correlation with SCP in the nasal quadrant (*p* = 0.016, r = − 0.445) and with all DCP regions [DCP superior (*p* = 0.004, r = − 0.519), DCP inferior (*p* = 0.004, r = − 0.519), DCP nasal (*p* = 0.006, r = − 0.496), DCP temporal (*p* = 0.005, r = − 0.508), DCP CSF (*p* < 0.001, r = − 0.634)]. The patients’ age or the attack frequency did not correlate with any of the examined parameters.

**Table 1:** Macular microvasculature and optic nerve head parameters of the dominant side of patients with episodic migraine compared to the non-dominant side and to healthy volunteers.

	Healthy volunteers§	P1*	Patients with migrainedominant side§	P2*	Patients with migrainenon-dominant side§
VD of SCP superior (%)	50.715 ± 2.474(49.694–51.736)	0.237	49.583 ± 2.379(48.695–50.472)	0.975	49.594 ± 2.947(48.494–50.695)
VD of SCP inferior (%)	50.778 ± 3.709(49.420–52.399)	0.108	48.927 ± 2.526(47.977–49.816)	0.465	49.342 ± 2.798(48.370–50.453)
VD of SCP nasal (%)	46.240 ± 2.340(45.342–47.183)	0.872	46.160 ± 1.555(45.559–46.753)	0.371	45.689 ± 3.350(44.522–47.103)
VD of SCP temporal (%)	47.355 ± 2.200(46.487–1.639)	0.163	46.651 ± 1.834(45.876–47.379)	0.064	47.406 ± 2.020(46.634–48.264)
VD of SCP CSF (%)	22.601 ± 3.376(21.238–23.872)	0.01*	19.980 ± 3.757(18.562–21.349)	0.682	19.843 ± 4.090(18.235–21.450)
FAZ area (µm^2^)	247.124 ± 75.991(218.745–276.988)	0.04*	307.366 ± 88.129(273.998–341.774)	0.194	306.607 ± 114.150(262.344–350.870)
VD of DCP superior (%)	51.819 ± 3.596(50.438–53.193)	0.765	52.246 ± 2.694(51.262–53.256)	0.657	52.385 ± 3.212(51.175–53.585)
VD of DCP inferior (%)	52.418 ± 2.760(51.336–53.555)	0.587	51.706 ± 2.577(50.709–52.704)	0.04*	53.233 ± 3.944(51.724–54.819)
VD of DCP nasal (%)	49.014 ± 2.689(47.884–50.044)	0.359	48.180 ± 2.945(47.071–49.330)	0.459	48.038 ± 3.352(46.741–49.345)
VD of DCP temporal (%)	46.933 ± 2.746(45.767–47.978)	0.522	47.517 ± 2.438(46.538–48.372)	0.023*	48.943 ± 2.578(47.979–49.980)
VD of DCP CSF (%)	19.273 ± 4.123(17.680–20.846)	0.004*	15.770 ± 3.291(14.614–16.996)	0.361	15.828 ± 3.434(14.392–17.196)
GCL + CSF (µm)	53.000 ± 8.124(49.830–56.280)	0.042*	47.070 ± 9.719(43.470–50.740)	0.160	47.800 ± 7.724(44.750–50.920)
GCL + inner ring (µm)	92.790 ± 7.235(89.945–95.353)	0.310	90.343 ± 6.166(88.060–92.595)	0.449	90.160 ± 6.204(87.491–92.452)
GCL + outer ring (µm)	68.800 ± 7.046(66.181–71.809)	0.306	67.164 ± 4.662(65.423–68.935)	0.138	67.660 ± 3.995(66.095–69.208)
GCL + + CSF (µm)	60.360 ± 8.925(56.820–63.970)	0.029*	53.190 ± 11.327(49.19–57.43)	0.281	54.600 ± 9.269(51.150–58.420)
GCL + + inner ring (µm)	118.170 ± 9.149(114.445–121.453)	0.327	115.028 ± 7.285(112.334–117.657)	0.046*	115.640 ± 6.896(112.823–118.173)
GCL + + outer ring (µm)	107.860 ± 10.041(103.784–111.272)	0.710	108.593 ± 6.429(106.303–111.200)	0.368	108.940 ± 5.957(106.578–111.261)
RNFL superior (µm)	135.480 ± 18.026(128.660–142.960)	0.532	133.630 ± 14.781(128.200–139.420)	0.737	131.64 ± 14.978(125.930–137.250)
RNFL inferior (µm)	140.320 ± 16.157(134.410—146.910)	0.716	139.850 ± 14.419(134.690–145.540)	0.201	140.000 ± 11.240(135.810–144.190)
RNFL nasal (µm)	84.200 ± 14.416(78.840–90.370)	0.748	84.890 ± 10.899(80.920–89.520)	0.135	83.08 ± 11.543(78.570–87.680)
RNFL temporal (µm)	81.480 ± 12.248(76.87–8.888)	0.021*	75.150 ± 9.130(71.58–78.500)	0.500	73.920 ± 9.954(70.150–77.960)
RNFL total (µm)	110.440 ± 10.890(106.420–115.100)	0.322	108.440 ± 8.613(105.160–111.790)	0.180	107. 240 ± 8.115(104.170–110.220)
BMO (µm)	1717.280 ± 142.988(1659.570–1774.500)	0.510	1688.890 ± 156.814(1634.580–1749.140)	0.110	16.24.640 ± 172.515(1563.710–1688.520)
BTE (µm)	1872.760 ± 169.587(1803.110–108.583)	0.035*	1780.330 ± 172.363 (1720.770–1848.050)	0.096	1737.600 ± 182.130(1671.600–1807.450)
ASO (µm)	1521.640 ± 163.787(1456.610–1596.190)	0.754	1538.590 ± 147.634(1489.190–1596.520)	0.077	1505.320 ± 166.400(1447.400–1570.720)
ONHRA (mm^2^)	1.472 ± 0.364(1.328–1.619)	0.606	1.470 ± 0.353(1.341–1.616)	0.198	1.494 ± 0.352(1.360–1.636)
DA (mm^2^)	1.905 ± 0.404(1.748–2.079)	0.729	1.940 ± 0.394(1.797–2.084)	0.946	1.914 ± 0.357(1.784–2.055)
lCDR	0.430 ± 0.188(0.354–0.505)	0.953	0.440 ± 0.209(0.356–0.511)	0.895	0.436 ± 0.160(0.370–0.497)
vCDR	0.414 ± 0.181(0.340–0.485)	0.939	0.419 ± 0.202(0.338–0.489)	0.328	0.430 ± 0.159(0.366–0.491)
CV (mm^3^)	0.053 ± 0.057(0.029–0.077)	0.506	0.076 ± 0.084(0.044–0.107)	0.405	0.067 ± 0.089(0.035–0.102)

*VD*, vessel density; *SCP*, superficial capillary plexus; *DCP*, deep capillary plexus; *FAZ*, foveal avascular zone; *GCL*, ganglion cell layer; *RNFL*, retinal nerve fiber layer; *CSF*, thickness within central 1 mm; inner ring, thickness within central 3 mm; outer ring, thickness within central 6 mm, *BMO,* Bruch´s membrane opening; *ASO*, anterior scleral canal opening; *BTE,* border tissue of Elschnig; *ONHRA,* Optic nerve head rim area; *DA,* disc area*; lCDR,* linear cup-to-disc ratio; *vCDR,* vertical cup-to-disc ratio; *CV*, cup volume.

§ Mean ± standard deviation (95% confidence interval).

P1 Mann–Whitney U test, healthy volunteers versus dominant side of episodic migraine patients, P2 Wilcoxon test, dominant side versus non-dominant side of episodic migraine patients.

*P ≤ 0.05.

Evaluating optic nerve head parameters, RNFL thickness in the temporal quadrant (*p* = 0.021) and BTE (*p* = 0.035) showed a significant decrease (Fig. 3), while no difference was found in the RNFL thickness in the other 3 quadrants or among further ONH parameters (BMO, BTE, ONHRA, DA, lCDR, vCDR, and CV; Table 1).

**Fig. 4:**
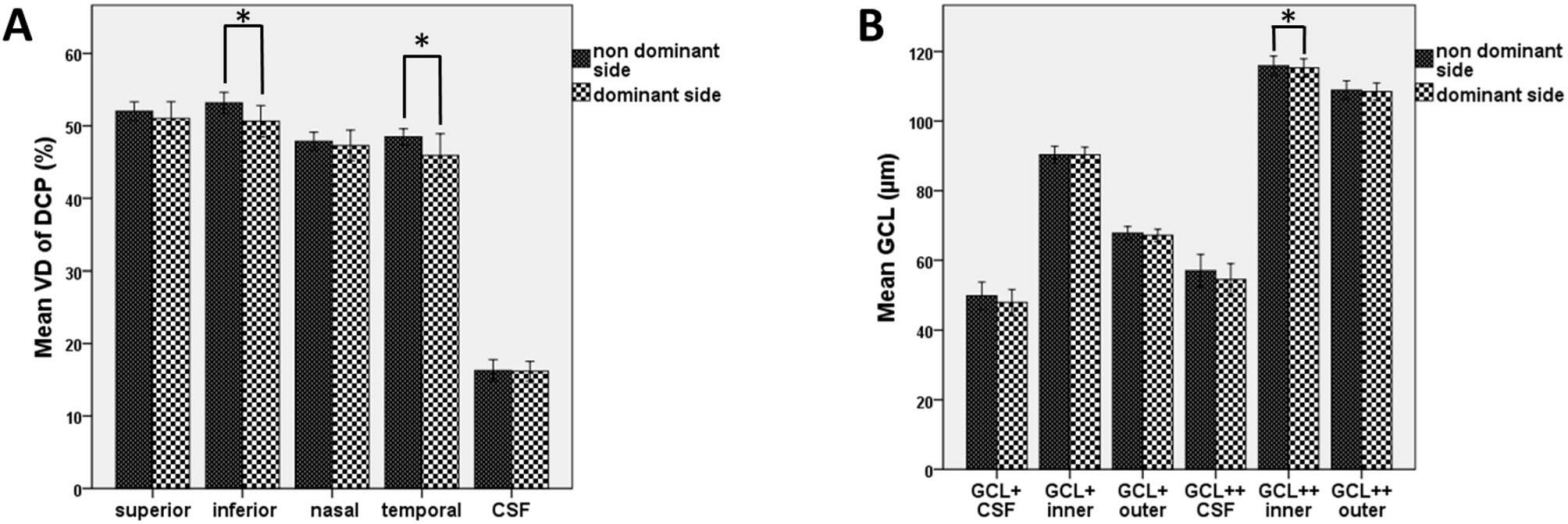
Changes in macular parameters observed on the dominant side of episodic migraine patients compared to the non-dominant side. (**A**) significantly reduced VD of DCP in the inferior and temporal quadrants; (**B**) significantly decreased thickness of the GCL +  + inner zone (*p ≤ 0.005).

Analyzing the difference between the dominant and non-dominant side of migraine patients a significant deterioration of the following parameters could be explored on the dominant side (Table 1). The vessel density was significantly reduced on the dominant side in the inferior (*p* = 0.04) and temporal quadrants (*p* = 0.023) of the DCP and showed a tendency to decrease in the temporal quadrant of the SCP (*p* = 0.064) (Fig. 4). Furthermore, the thickness of GCL +  + inner ring showed a significant decrease (*p* = 0.046; Fig. 4). Regarding the optic nerve head parameters, no dissimilarity could be found (Table 1).

Analyzing the non-dominant side of migraine patients compared to controls, a milder, but significant decrease of the VD was observed in the central zone of SCP (*p* = 0,012) and DCP (*p* = 0,011), and correspondingly the foveal avascular zone was significantly enlarged (*p* = 0,022). Nevertheless, without structural consequences, GCL + and GCL +  + showed no statistically significant changes on the non-dominant side of migraine patients compared to controls.

We evaluated the dominant side of migraine patients having an aura compared to the dominant side of the patients not suffering from auras and found no significant alteration in any of the examined parameters. Similarly, no difference could be detected when assessing the dominant side of patients with and without WMHI or the 3 subgroups defined based on the frequency of migraine attacks or the duration of migraine.

## Discussion

Recent development in OCT technology has allowed fast, non-invasive and detailed visualization of the posterior segment, encompassing the vascular dysregulation in the eyes of migraine patients. Previous evidence suggests that altered retrobulbar circulation plays a role in the etiology of migraine, probably due to the higher resistance in the central retinal artery and posterior ciliary artery during a migraine attack or headache-free period^14^. A case report of a patient during a migraine attack with visual aura showed narrowing of the retinal vessels and decreased radial peripapillary capillary density, superficial and deep foveal vessel density^15^. Consequently, the transient and recurrent decrease in blood supply to the ONH may lead to ischemic ganglion cell death^16^. Accordingly, we found a significantly decreased vessel density in the central zone of the superficial and deep capillary plexus of our episodic migraine patients. Corresponding to reduced circulation, a significant decrease in the thickness in the central zone of the ganglion cell layer (GCL + , GCL + +) could be documented. We could also confirm the presence of the significantly enlarged FAZ, as reported previously in migraine with aura patients^7^. Romozzi et al. suggested that the microangiopathy affecting the retina might originate from perpetrated microvascular insult over time^7^. Our finding, that the duration of migraine showed an inverse correlation with the VD of DCP in all quadrants and with the VD of SCP in the nasal quadrant support this concept. Comparable to our outcome, Altunisik et. al. did not find a significant difference between OCT parameters of migraine patients with and without WMH or aura either^17^.

Former studies suggest the importance of the lateralization of migraine headache in connection with pathological changes in the visual system. Gunes et al. investigated the relationship between migraine headache lateralization and RNFL thickness in migraine patients with unilateral headache. They found that the average and nasal RNFL thicknesses were significantly thinner on the side of headache and on the contralateral side compared to control eyes. Thinning was greater on the side of headache, but it did not reach significance^18^. Furthermore, interhemispheric differences of fMRI responses to visual stimuli in patients with side-fixed migraine aura have been described even in the interictal phase^19^. The transitory cerebral vasospasm leading to the diminution of blood supply which occurs before or during the onset of pain, is frequently localized to the posterior area of one hemisphere^20^. Our results suggest for the first time the presence of significant reduction in macular vessel density on the dominant side not only compared to controls, but to the contralateral side.

The intraocular part of the optic nerve is 1.0 mm in length and extends from the surface of the optic disc to the posterior margins of the sclera. With the latest advances in OCTs using a swept source technology, besides the quantitative assessment of the superficial NFL thickness we are able to quantify the optic nerve head morphology parameters. Perfusion changes (hypo-/hyperperfusion) during attacks may be limited to or begin in various brain regions or even outside the brain, e.g. in the retinal and choroidal circulation. Nonetheless hypoperfusion of cerebral and retrobulbar arteries is a transitory event, the chronicity and repetitive occurrence is assumed to result in permanent cerebral and retinal damage^21^. Moreover, migraine has been proposed to be risk factor for ischemic optic neuropathy and normal tension glaucoma^22^.

Regarding pre-scleral neural canal parameters, we found significantly lower BTE values on the dominant side of migraine patients compared to healthy controls. Sahan et al. detected optic nerve hardening in migraine compared to healthy controls using the quantitative elastic modulus of the optic nerve. They presumed that the increased optic nerve hardness might be caused by fibrotic changes associated with stiffening of the optic nerve. They also described a lower optic nerve sheath’s mean diameter in migraine compared to controls (5.04 ± 0.97 mm versus 5.19 ± 0.84 mm, *P* > 0.05)^23^.

We found a significant reduction of the temporal RNFL thickness in migraine patients on the dominant as well as on the contralateral side. Changes in the RNFL of migraine patients have been extensively investigated with spectral domain OCT; however, data from different studies are not homogeneous. Martinez et al. described the significant reduction of temporal RNFL thickness in migraine patients similarly to our results^24^. Reduction of peripapillary retinal nerve fiber layer (pRNFL) thickness in the temporal or in the upper optic nerve quadrants that have been reported by several authors, but was not confirmed by others [reviwed in 7].

To the best of our knowledge, this is the first study to describe retinal microvascular changes in association with the lateralization of migraine headache. The ultimate aim is to identify retinal biomarkers as objective indicators of episodic migraine. However, longitudinal studies are necessary to filter out patients with an elevated risk of developing serious systemic complications.

## Data Availability

Research data are available from the corresponding author on reasonable request.
